# The Uncertain Certainty: A Mixed Methods Exploration of Personal Meanings of Death and Preliminary Insights Into Their Relationship With Worldview

**DOI:** 10.1177/00302228231157135

**Published:** 2023-02-13

**Authors:** Daniel Spitzenstätter, Tatjana Schnell

**Affiliations:** 1 Existential Psychology Lab, Institute of Psychology, 27255 University of Innsbruck, Innsbruck, Austria; Existential Psychology, Social Sciences, MF Norwegian School of Theology, Religion, and Society, Oslo, Norway

**Keywords:** death, meaning, religiosity, spirituality, atheism, agnosticism, mortality, worldview

## Abstract

The present mixed methods study investigated personal meanings of death, i.e., concepts, views, and expectations associated with one’s own death, and explored their relation to worldview. To this end, a sample of 202 young, German-speaking adults completed the *Death Statements Test*, a new qualitative assessment tool, as well as quantitative measures of religiosity, spirituality, atheism, and agnosticism. Qualitative data was transformed to enable quantitative analyses. Results indicated that the spectrum of personal meanings of death is generally broad and multifaceted. The most prevalent view on death was “death as source of motivation and meaning in life.” The frequencies of emotionally positive and negative death meanings were relatively balanced, while neutral statements dominated. Relationships between participants’ death meanings and worldview dimensions turned out to be small but existent. The *Death Statements Test* proved to be a valuable and economic assessment tool, eliciting rich qualitative material on personal meanings of death.

  So death is indefinable—the only certainty, and the only thing about which nothing is certain.

—Søren Kierkegaard, *At a Graveside*

## Introduction

According to Søren Kierkegaard, a Danish religious writer and philosopher, the *earnest* thought of one’s own death “gives life force as nothing else does; it makes one alert as nothing else does” (Kierkegaard, 1993, p. 83). Contrasting Epicurus’ view that death “is nothing to us” (Diogenes Laertius, 2018, p. 534), he emphasizes the pivotal role of an individual’s personal stance toward their own death for living a truly authentic and meaningful life. From an existential-psychological perspective, a thorough, empirical exploration of this personal stance(s) toward death thus constitutes a worthwhile endeavor. This raises the question of what concepts, views, or expectations individuals subjectively associate with their own mortality—or: what specific, *personal meanings* they attribute to their own death.

It can be assumed that such personal meanings of death are—at least partially—associated with a person’s worldview orientation. Interestingly, very little is known yet about the relationship between an individual’s subjective understanding of death and their worldview. This is especially true for non-religious worldviews, as, for example, atheism, agnosticism, or “spirituality-without-religion.” During the last decades, all of these have established their place in the landscape of worldview orientations in Western societies (see, e.g., Keysar & Navarro-Rivera, 2013; Pew Research Center, 2018; Voas, 2008; WIN-Gallup International, 2012). The present study pursues two main objectives: First, to develop and test an improved assessment tool to investigate personal meanings of death. And second, to explore the relationship between these death meanings and an individual’s worldview orientation.

According to Taves et al. (2018, p. 208), worldviews can be understood “as a complex set of representations related to ‘big questions’”, such as ontology, epistemology, axiology, praxeology, and cosmology, “that define and govern a way of life.” Because this set of representations is hard to grasp empirically in its entirety, we focus on the question of whether God, gods, or any other supernatural entities exist or not. The belief in such transcendental powers typically includes the promise of “literal immortality”, a cross-culturally shared strategy to conquer the dread of death (Becker, 1973; Solomon et al., 2004). We found this aspect of an individual’s worldview particularly interesting with regard to the study of personal meanings of death. In the following, we use “worldview” as an umbrella term for religiosity, spirituality, agnosticism, and atheism—four different answers to this fundamental question.

### Personal Meanings of Death

*Personal Meanings of Death* can be understood as subjective attitudes or stances toward the objective factum of one’s own mortality (Cicirelli, 1998). They are “primarily cognitive interpretations of objects and events associated with death that are derived from the individual’s experience” (Cicirelli, 2001, p. 663) and can be either positive (e.g., comforting, reassuring), negative (e.g., frightening), or even neutral (or indifferent) with regard to their emotional valence (see Durlak et al., 1990). As several authors have noted, personal meanings of death are in a dynamic and mutual relationship with personal goals, values, and orientations in life (Cicirelli, 2001; Mikulincer & Florian, 2008; Tomer, 2000; Wong et al., 1994). Cicirelli (2001) further argued that personal meanings of death also affect how an individual encounters the issues of death and dying:

Personal meanings [of death] may influence the way a given individual lives, the way in which he or she reacts to death, the fears of death that he or she might have, conceptions about the dying process, and the way that he or she prepares for death. (p. 664)

Several quantitative instruments are available to directly or indirectly assess personal meanings of death (e.g., Abdel-Khalek, 2002; Cicirelli, 1998; 2001; Florian & Kravetz, 1983; Hoelter, 1979; Spilka et al., 1977). However, significant shortcomings regarding their psychometric properties (e.g., factorial structure, problematic cross-loadings) or their usability with non-religious samples (e.g., wording and interpretability of items) have to be stated. Moreover, and although the questionnaires vary substantially regarding the number of constructs they comprise, the assessable scope of death meanings is limited due to the full standardization of these instruments. The researcher typically does not know whether their predefined questions really cover the *subjectively most relevant or salient* meanings a participant associates with their death (Durlak et al., 1990; Holcomb et al., 1993).

From this perspective, qualitative free-response formats to assess personal meanings of death have considerable benefits: First, they are open for an individual’s very own views regarding death—rather than limited to several preselected constructs. Second, biases through specific wordings or due to a long series of direct, death-related questions are mitigated. Third, the salience of certain death meanings can easily be assessed—simply by their occurrence (or non-occurrence) in participants’ responses (see Durlak et al., 1990; Holcomb et al., 1993).

Probably the most versatile qualitative instrument in this regard was developed by Neimeyer et al. (1983) and later slightly expanded by Holcomb et al. (1993). With its 15 content categories, the *Death Construct Coding Manual* is a comprehensive tool to analyze any death-related text material. However, in our view, several potential—especially interpersonal—death meanings seem to be missing in that list. Another promising qualitative approach to assess personal meanings of death was presented by Durlak et al. (1990). They employed an adapted form of the *Twenty Statements Test* (Kuhn & McPartland, 1954) and analyzed the obtained text material along seven, relatively broad content categories.

In the present study, we tried to combine the economic approach to gather qualitative data on death meanings used by Durlak et al. (1990) with a comprehensive and detailed content analysis inspired by Neimeyer et al. (1983). We developed the *Death Statements Test* (DST), an improved approach to assess personal meanings of death, which will be described more detailed in the Method section. In order to put our own findings in a broader context, we conducted an extensive synopsis with previous research programs on personal death meanings. This comparison and evaluation will be presented and summarized in the Discussion.

### Psychological Research on the Relation between Worldview and Death Attitudes

For the most part, psychological research on death attitudes has focused on anxiety and fears related to death. To a lesser degree, also positive attitudes, i.e., death acceptance, were investigated (Neimeyer et al., 2004). Empirical findings on broader conceptions of death attitudes, i.e., personal meanings of death, and their relation to worldview are currently scarce to non-existent. The following overview will thus focus on death anxiety and death acceptance, which are expected to be linked in multiple ways to personal meanings of death (see above).

#### Religiosity and Attitudes Toward Death

Although the question of how religiosity relates to death anxiety has a long history in thanatological theory and research, empirical results were often inconsistent and inconclusive (Jong et al., 2018). As a growing body of empirical evidence now suggests, both might be related curvilinearly (rather than linearly), representing an inverted U-shape. That means that highly religious and highly *non*-religious individuals are less anxious about their death than the moderately (non-)religious (for reviews, see Ellis & Wahab, 2013; Jong et al., 2018; Wink & Scott, 2005). A recent study by Spitzenstätter and Schnell (2020) expanded this finding by showing that the moderately (non-)religious are not only more fearful but also more avoidant toward their death. As several authors have argued, these results suggest that personal conviction and certainty in respect of one’s worldview might be more relevant concerning death anxiety (and avoidance) than the actual content of (religious or non-religious) beliefs (Ellis & Wahab, 2013; Jong et al., 2018; Neimeyer et al., 2004; Sawyer et al., 2019; Spitzenstätter & Schnell, 2020; Wink, 2006; Wink & Scott, 2005).

Besides the negative attitudes of death anxiety and avoidance, some research was also undertaken to examine the relationship between religiosity and a more positive attitude toward death, namely death acceptance. Since different approaches for measuring and conceptualizing this construct have been proposed (see Neimeyer et al., 2004; Wittkowski, 2001; Wong et al., 1994), results are often difficult to compare. Employing the Three-Component Model of Death Acceptance by Wong et al. (1994), several studies found that religiosity, especially when intrinsically motivated (Allport & Ross, 1967), is highly positively correlated with the belief in a happy afterlife (= approach acceptance). In addition, intrinsic religiosity was moderately positively correlated with the view of death as a relief from pain and suffering in life, termed escape acceptance, but not with the conceptualization of death as a natural and integral part of life, i.e., neutral acceptance (Ardelt, 2008; Lin, 2003; Tomer & Eliason, 2005). Spitzenstätter and Schnell (2020) also found a strong positive relationship between religiosity, operationalized as religious life and personal relationship with God, and approach acceptance. In contrast to the studies cited before, however, they found a small negative relationship between religiosity and neutral acceptance but none with escape acceptance.

#### Secularity and Attitudes Toward Death

Studies researching death attitudes in relation to secular worldviews like atheism and agnosticism are still an exception. The few published studies also have their pitfalls: Lundh and Radon (1998), as well as Feldman et al. (2016), surveyed religious and secular individuals (atheists, agnostics) on death anxiety and other death attitudes. Apart from yielding conflicting results, at least for death anxiety, the main shortcoming of these two investigations lies in the assignment to the different belief groups, operationalized through one simple yes/no(/uncertain)-question. Sawyer et al. (2019) investigated death attitudes in a sample of self-identified non-believers but did not analyze their results for different forms of non-belief (e.g., atheism, agnosticism). Spitzenstätter and Schnell (2020) assessed atheism and agnosticism dimensionally—instead of employing a categorical assignment to one of several groups—and investigated linear and curvilinear relationships between these worldview dimensions and different death attitudes. They found that atheism showed the same curvilinear relationship to death anxiety and death avoidance as religiosity, i.e., individuals low or high in atheism were less fearful and avoidant than the moderately atheistic. In addition, atheism exhibited a small to moderate (linear) positive relationship with neutral acceptance, a high negative relationship with approach acceptance, and no association with escape acceptance. Agnosticism, in contrast, was (linearly) positively related to death anxiety, death avoidance, and approach acceptance, negatively related to escape acceptance (all effect sizes were small to modest), but not related to neutral acceptance.

#### Spirituality and Attitudes Toward Death

As far as spirituality is understood as a construct distinct from religiosity, in the sense of a more individualistic, open approach to a higher reality (e.g., Schnell, 2012; 2019; Zinnbauer et al., 1997), empirical findings on associations with death attitudes are rare to non-existent. Spitzenstätter and Schnell (2020) found that spirituality showed a small to moderate negative relationship with neutral acceptance, was highly positively correlated with approach acceptance, but was not significantly associated with escape acceptance. Concerning death anxiety and death avoidance, no statistically significant relationships were observed—neither linearly nor curvilinearly. An earlier study by Wink (2006) also found that spirituality was not related to death anxiety but moderately positively correlated with approach acceptance.

### The Present Study

The present study aimed to explore personal meanings of death. Due to the mentioned limitations of quantitative instruments, death meanings were assessed by employing an open, qualitative tool. In this way, it was possible to investigate a spectrum of meanings as broad as possible and to consider interindividual differences in the level of importance participants ascribe to certain concepts, views, or expectations related to their death. The elicited qualitative data was then content analyzed, and distinct categories representing specific death meanings were formed. The study’s second aim was to relate the presence of personal meanings of death to a participant’s worldview, which was assessed quantitatively. We employed established psychometric instruments for measuring the degree of religiosity (= a personal relationship with God and religious life), spirituality (= a subjective approach to a higher reality), atheism (= the denial or lack of belief in the existence of God/a higher power), and agnosticism (= the view that the metaphysical question of God/a higher power is unanswerable). This multi-dimensional approach allowed to capture an individual’s worldview more comprehensively than a mere categorical (self-)assignment to one of several belief groups (see, e.g., Bullivant, 2008; Cragun, 2019; Galen & Kloet, 2011; Schnell, 2015).

The following research questions guided the study: i. What meanings do individuals associate with their own death? ii. What are the most prevalent meanings in this regard? iii. Is the presence of specific death meanings in an individual related to their level of religiosity, spirituality, atheism, or agnosticism? The study was reviewed by the University of Innsbruck’s *Board for Ethical Issues* (approval number: 21/2020) and granted ethical clearance.

## Method

### Qualitative Part: Death Statements Test (DST)

To elicit specific meanings individuals associate with their death, we employed an improved assessment tool, based on the *Twenty Statements Test* (TST; Kuhn & McPartland, 1954). The TST was initially developed to study a person’s self-concept by giving respondents a stimulus question (typically: “Who am I?”) and asking them to fill in their answers to this question into 20 blank lines. These twenty self-definitions subsequently constitute the coding units for content analysis. The TST is unique in its combination of maximum response openness, eliciting rich qualitative material, with a highly structured and quantifiable form of assessment (see Rees & Nicholson, 2004). Moreover, through its indirect way of assessment, the TST can provide information about the *salience* of specific attitudes and concepts, which is a crucial advantage over direct questions used in fully standardized, quantitative instruments (Kuhn & McPartland, 1954).

The TST has been used to study death attitudes at least twice: First by Bakshis et al. (1974), and second by Durlak et al. (1990). Both authors used different stimulus questions (“What is death?” and “What does your death mean to you?”, respectively) as well as different coding strategies; neither of them related their results to their participants’ worldview. An instrument based on a TST-approach seemed most suitable to explore not only participants’ fears and hopes related to death, but a wide spectrum of idiosyncratic death meanings.

To elicit the death meanings most relevant to the individual, we changed the probe question for the present study into “What does my death mean to me?”, which is closer to the original stimulus and the initial intention of encouraging participants to give their answers as if they were giving them to themselves (Kuhn & McPartland, 1954). To test our adaption of the TST, we conducted a pilot study with 26 participants. One conclusion drawn from this pretest was that most participants were unable to formulate as many as 20 statements related to their death (*Mdn* = 11, mode = 10, range = 6–20). Therefore, and in line with the procedure of Bochner (1994), the maximum number of answers was restricted to ten in the present study; each case with at least seven meaningful responses was counted as a valid test result. An additional insight drawn from the pretest was that participants should be instructed to give their answers in complete sentences rather than single words, as this increased interpretability. Furthermore, participants were instructed to work on the task without interruptions, fill in the first and most spontaneous associations that come to their mind, and write their statements down without hesitation. Due to the reported adaptions and in order to avoid confusions, we refer to our assessment tool as *Death Statements Test* (DST) (the full DST is available as Supplemental Material A).

### Quantitative Part

Four dimensions of an individual’s worldview were assessed: religiosity, spirituality, atheism, and agnosticism. Religiosity (“Religion plays an important role in my life”, 3 items, α = .91) and spirituality (“There are certain things in life I consider sacred”, 5 items, α = .72) were operationalized by the respective subscales of the SoMe-questionnaire (Schnell, 2014; 2021; for the German version, see Schnell & Becker, 2007). To measure individual levels of atheism and agnosticism, we employed the corresponding scales of the DoS-inventory by Schnell (2015). The internal consistencies were α = .90 for atheism (“There is no such thing as a god/a higher power”, 5 items) and α = .89 for agnosticism (“There might be a higher power/a god, but we will never know for sure”, 5 items). For all four dimensions, a 6-point Likert scale, ranging from (0) *do not agree at all* to (5) *agree completely*, was used and items were averaged.

Additionally, a demographical questionnaire assessed participants’ age, gender, nationality, education, employment status, and their belonging to a religious community.

### Participants and Procedure

From May to June 2020, 202 persons participated in the study, which was implemented using the online survey tool *So*S*ci Survey* (Leiner, 2019). A convenience sample was recruited via social media and a university newsletter. As a trade-off between the highly time-consuming qualitative content analysis and considerations regarding statistical power, we envisaged a sample size of about 200 participants. The study was advertised as a psychological investigation of attitudes toward life and death. To inhibit any priming effects regarding one’s own worldview (which in turn could influence a participant’s responses to the DST), no references to terms like “religiosity,” “spirituality,” or “secularity” were made. Inclusion criteria were a minimum age of 18 years, provided consent, and completion of the entire online survey. Among all participants, 150 were female, 50 were male, and two identified as non-binary. The sample’s mean age was 26 years (*SD* = 7, *Mdn* = 24, range: 18–57); 72% were university students. The majority of the participants were Austrian (48%) or German (39%), others were Italian (10%) or other Europeans (4%). With 46% of the study’s partakers holding a bachelor’s degree or higher, the educational level was relatively high. Of those who reported belonging to a religious community (68%), all were Christian (Roman-Catholic: 80%; Protestant: 17%; other: 2%), except for one Buddhist (1%).

After providing informed consent, participants first completed the DST (qualitative), then the assessment regarding their worldview (quantitative), and lastly, the demographical questionnaire (quantitative). Mean time for completion of the DST was 6.38 minutes (*SD* = 4.56).

## Data Analysis

### Qualitative Analysis

The first author analyzed the qualitative data gathered with the DST using qualitative content analysis (QCA), following Mayring (2015). Each given statement in the DST (from a minimum of 7 to a maximum of 10) represented one unit of analysis and was coded with one exclusive content category. Content categories were derived as follows: First, an initial category system was built inductively by paraphrasing, generalizing, and reducing 10% of the text material (see Mayring, 2015). The category system was then iteratively improved by applying it in steps of 10% to the remaining material. After 100% of the material was analyzed, the category system was described in detail in a coding manual.

In addition to the content of the text material, the first author rated the emotional valence of each statement (following Durlak et al., 1990). This rating tried to capture the participants’ subjective (rather than a socially shared or “objective”) interpretation of a specific concept or expectation and was coded either as *positive*, *neutral*, or *negative*. Examples for each of the three rating values are “My death means gratitude for me.” (positive), “Death is a necessary part of life.” (neutral), and “Death renders my life meaningless.” (negative).

After the first version of the coding manual was completed, the first author and two additional independent raters applied the category system again to the entire text material. The independent raters were graduate students in psychology with experience in QCA, were not involved in the research project from which the present study originated, and were trained by the first author in the use of the coding manual. For this training, about 200 statements not related to the current study were used to practice the coding procedure. Some coding rules and instructions within the manual got slightly adapted and refined to eliminate ambiguities or uncertainties raised by the independent raters (version 2 of the manual). When both independent raters were confident in using the manual, all three raters independently coded all statements from the study material. (*Note.* For a summary of the final version of the coding manual in English language, see Supplemental Material B. The full version in German language is available from the first author upon reasonable request.)

To determine the agreement between raters, i.e., the inter-rater reliability of the category system, we calculated Krippendorff’s alpha values (α_K_; Hayes & Krippendorff, 2007). Krippendorff (2004) recommends relying only on variables with reliabilities above α_K_ = .80. The reliability values for both content (α_K_ = .83) and emotional valence ratings (α_K_ = .82) of the three raters fulfilled this criterion, thus implying good inter-rater reliability of the category system. To examine the agreement within raters, i.e., the intra-rater reliability of the category system, all three raters coded a subset of 25% of the study material (randomly selected) again after 4 weeks, blind to the results of their first codings. The agreements between their two ratings were above the threshold of α_K_ = .80 in each case (ranging from α_K_ = .84 to .92) and thus indicate sufficient intra-rater reliability of the category system.

After determining reliability values, cases of disagreement between the three raters were resolved according to the majority vote, when two out of three raters agreed upon a content (19% of statements) or emotional valence (19%) rating. When all three raters disagreed on a statement’s content or emotional valence rating (3% and 0.05%, respectively), it was resolved consensually.

### Transformation of Qualitative Data

To enable quantitative analyses of qualitative data, the following variables were computed:

#### Presence (individual level)

The presence of a specific death meaning was operationalized as a dichotomous variable. It was coded as “1” if one or more of the participant’s statements corresponded to this content category and as “0” if not.

#### Emotional Valence (individual level)

Three variables were computed on the relative frequency of each rating category of emotional valence. To this end, the number of positive, neutral, or negative statements, respectively, was divided by the participant’s total number of statements. Each of these three continuous variables thus ranged from 0.00 to 1.00.

#### Prevalence (sample level)

To quantify which death meanings were most prevalent in the total sample, the presence values were summed up and divided by the number of participants (*N* = 202). In this way, the resulting variable (presented in percent) gives information on how many participants made at least one statement that corresponds to the specific content category.

### Quantitative Analyses

All statistical analyses were run with IBM SPSS Statistics Version 26. To compute inter- and intra-rater reliabilities, the KALPHA-macro by Hayes and Krippendorff (2007) was used. All significance tests were run two-sided.

## Results

### Elicited Death Meanings

Of the 202 participants, 86% gave ten, 1% nine, 5% eight, and 8% seven responses to the DST (*M* = 9.65, *SD* = 0.90). In total, 1,950 statements were collected. Through qualitative content analysis, 33 different content categories were deduced from the text material. Two additional residual categories were formed for statements that were not assignable to any of the content categories (1.8% of all statements) or not codable regarding their content in general (0.2%). Table 1 gives an overview of content categories (incl. anchor examples); the coding manual describes all categories in detail (see Supplemental Material B).

**Table 1.** Anchor Examples and Prevalence Values of Content Categories.

Category		Anchor examples	Pr.
A. General discomfort with death			
1.	Fear of death (unspecified)	“I fear death.” “Death frightens many people.”	16
2.	Loss and transience	“Death means loss to me.” “I will be forgotten.”	24
3.	Uncertainty and uncontrollability (non-transcendental)	“Something that can happen anytime.” “My death means a total loss of control for me.”	36
4.	Other negative associations	“Death means despair.” “I associate something evil with it.”	30
B. General comfort with death			
5.	Salvation and relief	“My death could be a relief after a long disease.” “Death could be a way out.”	36
6.	Peace and calmness	“I find my peace.” “Eternal sleep awaits me.”	13
7.	Other positive associations	“My death means gratitude for me.” “I am not afraid of death.”	29
C. Social consequences of one’s own death			
8.	Separation from close persons	“Death means to me that I lose my loved ones.” “All relationships will end.”	21
9.	Inability to be there for others	“When I am no longer alive, I cannot be there for others.” “I forsake my family.”	7
10.	Inconveniences for others	“My death means grief for my relatives.” “My dependents have to pay the funeral costs.”	28
D. Encounters with death			
11.	Death awareness	“Something that has already come very close to me.” “I think about death every day.”	13
12.	Taboo, repression, avoidance	“I repress the thought of death.” “My own death is an issue I rarely deal with.”	14
13.	Wish for long life or immortality	“I hope that I will live for a long time.” “I do not want to die.”	25
E. Rational-analytical considerations on death			
14.	Death as something natural and universal	“Death is a necessary part of life.” “Everyone has to die someday.”	37
15.	Epicurean view or indifference	“In the end, my death does not affect me at all because I will never know that I am dead.” “My death means nothing to me.”	23
16.	Cosmic insignificance	“My death means not much to the universe as a whole.” “The world will keep turning normally after my death.”	8
F. Individual life orientation and evaluation			
17.	Death as motivator or source of meaning	“The thought of my death reminds me of how important it is to live one’s own life.” “My death gives meaning to my life.”	41
18.	Death as a trial	“My death brings the reckoning for all my good and bad deeds.” “To draw a conclusion.”	6
19.	Termination of plans/goals/possibilities	“I can’t do anymore what I wanted to do but haven’t done yet.” “My death is the end of my possibilities.”	13
20.	Generativity and remembrance	“I do not want to die without having children.” “I want to be remembered.”	24
21.	Wish for a positive life review	“I want to have lived a fulfilled life.” “Hopefully, I do not regret anything.”	24
G. Postmortem (non-)existence			
22.	Death as the end (unspecified)	“Death is the end.” “Death means a closing for me.”	19
23.	Death as the end of physical functions	“The end of the vital functions of my body.” “My body will rot.”	16
24.	Death as the end of existence	“The end of my existence.” “I do not believe in a soul.”	28
25.	Death as the end of life	“The end of my life.” “Death means the loss of life.”	26
26.	Death as the end of mental abilities	“I cannot think anymore.” “I do not have thoughts or feelings anymore.”	7
27.	Belief in life/existence after death	“After death, there is life.” “My death is the beginning of another form of existence.”	19
28.	Postmortem uncertainty	“I am afraid that it won’t go on after death.” “I don’t know what will happen after death.”	27
29.	Hope for life/existence after death	“I hope for a life after death.” “I hope to see my dad again.”	16
H. Dying and the ending of life			
30.	Death as an experiential state	“Death means darkness for me.” “It means coldness for me.”	11
31.	Thoughts on funeral/burial	“I wonder who will attend my funeral.” “I want to be cremated after my death.”	11
32.	Thoughts on the dying process	“Will dying be painful?” “What do you think about when you are dying?”	19
33.	Wish for a “good” death	“I want to say goodbye to all my loved ones before I die.” “I want to die with dignity.”	20

*Note.* Pr. = prevalence (in percent).

**Figure 1.**
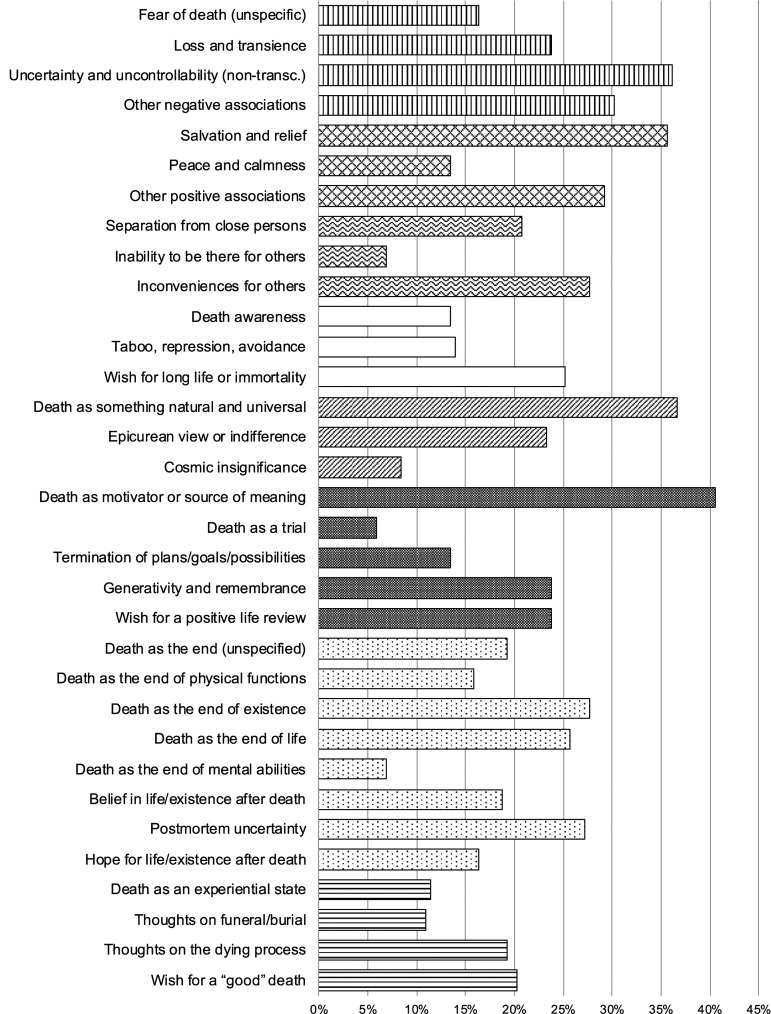
Prevalence of Qualitative Content Categories (Personal Meanings of Death). *Note.* Different patterns indicate different overarching themes (see Table 1).

For a better overview, thematically related content categories were grouped into eight broader, overarching themes (see Table 1 and Figure 1). As displayed in Table 1 and Figure 1, *death as motivator or source of meaning* (41%), *death as something natural and universal* (37%), *uncertainty and uncontrollability (non-transcendental)* (36%), and *salvation and relief* (36%) were the death meanings which were most prevalent among the surveyed participants. *Death as motivator or source of meaning* describes a view where individuals see death as a motivating force that influences the formation and realization of personal goals and priorities in life. Additionally, statements characterizing the thought on one’s own mortality as a trigger for viewing or evaluating life as something worthwhile and meaningful are covered by this category. Persons characterizing *death as something natural and universal* see death as a natural, necessary, and inevitable part of every human’s life. The category *uncertainty and uncontrollability (non-transcendental)* summarizes all statements that express uncertainty (e.g., regarding the time, place, or cause of one’s death) or uncontrollability related to death without an explicit reference to a hereafter or an existence after death (for which a separate content category was formed). When death is associated with *salvation and relief*, individuals typically refer to liberation from pain, suffering, stress, sorrows, daily obligations, or a meaningless life. Table 1 presents anchor examples for each content category.

Regarding the emotional valence of the DST statements, the averaged proportions of *negative* (*M* = 0.19, *SD* = 0.16) and *positive* (*M* = 0.16, *SD* = 0.17) statements were statistically equal (Wilcoxon signed-rank test, *Z* = −1.78, *p* = .076, *r* = −0.14), with the most statements rated as *neutral* (*M* = 0.65, *SD* = 0.21).

### Results Concerning a Participant’s Worldview

Based on descriptive values of the employed worldview measures, our sample can be described as rather secular. Agnosticism was most pronounced (*M* = 3.41, *SD* = 1.40), followed by atheism (*M* = 3.05, *SD* = 1.43), spirituality (*M* = 2.38, *SD* = 1.14), and religiosity (*M* = 1.13, *SD* = 1.39) (each ranging from 0 to 5).

#### Worldview and Presence of Death Meanings

As shown in Table 2, individuals with higher levels of religiosity were more likely than those with lower levels of religiosity to associate their death with various positive aspects (category: *other positive associations*), with a time of reckoning or a final reflection on one’s own life (*death as a trial)*, and with the *belief in life/existence after death*. Moreover, the religious were less likely to see death as something unimportant or indifferent to them (*Epicurean view or indifference*) and to view *death as the end of existence*. For atheism, the results mirrored those for religiosity in wide parts, i.e., significant correlations with opposite signs, with two exceptions: Those who endorsed atheism wrote more often than others about *inconveniencies for others* that their own death might entail, as well as that their own death feels insignificant when viewed from a neutral, impersonal point of view (*cosmic insignificance*).

**Table 2.** Correlations Between Worldview Dimensions and Personal Meanings of Death.

		Worldview			
Personal meanings of death		Rel.	Spir.	Ath.	Agn.
Content categories [presence]					
1. Fear of death (unspecified)		.04	.10	−.06	.12
2. Loss and transience		−.01	.06	.08	.09
3. Uncertainty and uncontrollability (non-transcendental)		.02	.04	−.07	.03
4. Other negative associations		.02	.11	−.13	**.24**
5. Salvation and relief		−.01	−.05	.09	−.04
6. Peace and calmness		−.13	−.10	.11	−.01
7. Other positive associations		**.19**	.12	**−.19**	−.00
8. Separation from close persons		.04	−.02	−.04	**.21**
9. Inability to be there for others		−.02	.10	.01	.04
10. Inconveniences for others		−.13	−.09	**.21**	−.04
11. Death awareness		.03	.01	−.02	−.04
12. Taboo, repression, avoidance		.01	−.02	−.02	.02
13. Wish for long life or immortality		−.03	−.08	−.03	.07
14. Death as something natural and universal		.10	−.05	−.06	−.05
15. Epicurean view or indifference		**−.19**	***−.17***	**.22**	−.09
16. Cosmic insignificance		−.09	***−.16***	***.16***	−.03
17. Death as motivator or source of meaning		−.02	−.03	−.03	−.06
18. Death as a trial		**.22**	.10	***−.17***	.10
19. Termination of plans/goals/possibilities		−.05	−.02	.12	.09
20. Generativity and remembrance		−.03	.07	−.04	.05
21. Wish for a positive life review		.01	.10	−.08	***.16***
22. Death as the end (unspecified)		−.09	−.07	.05	−.03
23. Death as the end of physical functions		−.04	−.10	.11	−.05
24. Death as the end of existence		**−.22**	**−.27**	**.23**	−.10
25. Death as the end of life		−.09	***−.14***	.11	.02
26. Death as the end of mental abilities		−.04	−.11	.12	−.04
27. Belief in life/existence after death		**.45**	**.37**	**−.46**	.02
28. Postmortem uncertainty		−.07	.04	.00	.10
29. Hope for life/existence after death		.09	***.15***	−.14	.11
30. Death as an experiential state		−.12	.02	.12	.04
31. Thoughts on funeral/burial		−.04	−.03	.10	−.11
32. Thoughts on the dying process		−.07	.00	.02	.00
33. Wish for a “good” death		−.05	.00	−.04	−.02
Emotional valence [relative frequency]					
−1	Negative	−.09	.09	.05	**.23**
0	Neutral	−.08	***−.17***	.08	***−.15***
+1	Positive	***.18***	.12	***−.14***	−.03

*Note.* Rel. = Religiosity; Spir. = Spirituality; Ath. = Atheism; Agn. = Agnosticism.

Pearson correlations; bold: significant at *p* < .01; bold and italic: significant at *p* < .05.

Spirituality was negatively related to viewing one’s own death as insignificant in a cosmic sense (*cosmic insignificance*), to an *Epicurean view or indifference* pertaining to death, and to viewing *death as the end of existence* or as the end of life (*death as the end of life*). Both the *belief in life/existence after death* and the *hope for life/existence after death* correlated positively with spirituality. Participants high in agnosticism were more likely to make statements in the DST that related to various negative associations with death (*other negative associations*), to a *separation from close persons* linked to one’s own death, or to the *wish for a positive life review* at the time of one’s own death than those low in agnosticism.

#### Worldview and Emotional Valence of Statements

As can be seen in Table 2, religiosity, as well as atheism, were unrelated to a negative emotional valence of statements made in response to the DST-stimulus, but both were associated significantly—and in different directions—to the number of positive statements made: Religiosity correlated positively and atheism negatively with positive emotional valence. In contrast, agnosticism correlated positively with negative emotionality but not with positive emotionality. Neutral emotional valence was significantly and negatively associated with spirituality and agnosticism but not with religiosity or atheism.

## Discussion

The present study sought to uncover meanings individuals associate with their death, and to explore relationships between those meanings and dimensions of worldview. Through qualitative content analysis, three independent raters elicited 33 different content categories, i.e., personal meanings of death, from text material based on responses to the *Death Statements Test* (DST). The death meanings spanned a wide spectrum of thematic areas, from fears and hopes to social and personal consequences, as well as metaphysical and normative positions related to (one’s own) death. The rich qualitative data elicited by the DST, i.e., the width of elicited death meanings, proved the usefulness of this assessment tool. Qualitative data was then quantified by calculating prevalence and presence values for each content category and relative frequencies for positive, neutral, and negative emotional valence ratings.

Results showed that the most prevalent death meanings were views of death as a motivating and meaning-creating force in life, death as something natural and universal, feelings of uncertainty and uncontrollability in the light of death, and expectations of salvation and relief accompanying death. Each of these associations was held at least by a third of all participants. With ca. 41%, the prevalence of *death as motivator or source of meaning* was the highest among the total sample. This finding is particularly interesting, given the often-claimed taboo and denial surrounding the topos of death in Western societies. The socio-cultural history and complexity of this issue is beyond the scope of the present study (for an overview, see Robert & Tradii, 2019; Tradii & Robert, 2019). However, our results suggest that a significant proportion of our participants considered a reflection of one’s own mortality as beneficial for their life, which at least calls the proposition of a universal and deep-rooted death denial in question.

Furthermore, our finding that participants made mostly emotionally neutral statements stands in opposition to an image of death as something primarily negative, dark, unwanted, highly feared or mostly avoided. In our study, it turned out that death is viewed—on average—rather calmly and neutrally. There *are* negative associations people attribute to their death, but they are also balanced by more positive views regarding one’s own mortality.

Notably, none of the elicited death meanings was reported by a majority of the participants. Taken together, we conclude that views and perspectives regarding one’s own death are highly individual and idiosyncratic. How can these interindividual differences be explained? It stands to reason that the attitude toward the existence of a deity or higher power is involved. We tested this assumption by correlating presence values of death meanings and the emotional valence of participants’ statements with the dimensions of religiosity, spirituality, atheism, and agnosticism.

Religious persons were likely to believe in life or existence after death and—accordingly—to not see their death as the end of their existence. They tended to associate death with some form of trial or reckoning and neglected an Epicurean or indifferent position on death. Religious individuals also reported more positive associations with death, which was exemplified not only through the connection with *other positive associations* (content category) but also by the correlation with positive emotional valence.

Spiritual individuals resembled religious persons in that they believed in—or at least hoped for—a life or another form of existence after death. They also described their death less often as the end of their existence or even as the end of their life. Furthermore, spirituality was inversely related to an Epicurean position on death and to view one’s own death as “cosmically insignificant”, i.e., when spirituality was high, one’s own death was not perceived as insignificant. Moreover, spirituality was unrelated to both negative and positive emotional valence. Its negative correlation with neutral emotional valence might indicate that higher spirituality is associated with more pronounced emotionality regarding death—either positive or negative. This finding ties in with Schnell’s (2012) observation that individuals with an open and idiosyncratic spirituality show higher levels of neuroticism than those holding a more traditional religious worldview—and even than the general population.

Atheists mirrored the profile of religious individuals by reporting fewer afterlife beliefs, describing death more often as the end of existence, holding an Epicurean view more frequently, not viewing death as a form of trial, and associating death less often with positive concepts (category *other positive associations*). However, atheists also showed a higher likelihood of associating death with inconveniencies for other persons. They were more likely to rate their death as insignificant from a cosmic point of view. Concerning emotional valence, atheism was associated with a less positive view on death, although that did not, in turn, result in a more negative view.

Whereas religiosity, spirituality, and atheism exhibited several similarities or opposites, agnosticism emerged as a relatively different worldview orientation regarding personal meanings of death: It did not correlate with any of those death meanings associated with either religiosity, spirituality, or atheism. Agnosticism was associated with a more negative view on death, which is evident by the significant positive correlation with negative emotional valence and a negative correlation with neutral emotional valence, as well as its connection with *other negative associations* with death (content category). Moreover, agnostics linked their death more frequently to a separation from close ones and more often made statements referring to a wish for a positive life review at the end of their days. These results support recent findings by Schnell et al. (2021), which suggested that agnosticism and atheism are clearly distinguishable secular attitudes.

Although moderate correlations between religiosity, atheism, and spirituality on one side and the belief in life or existence after death on the other side were observed, the effect sizes of most relationships between dimensions of worldview and personal meanings of death (including emotional valence) were around *r* = .20 or below—and therefore relatively small. This might be due to the high specificity of the investigated death meanings on the one hand; on the other hand, the finding supports the conclusion that an individual’s perspective on death is highly idiosyncratic. Finally, interindividual factors other than worldview might be relevant to the formation of death meanings too. Examples might include personal experiences with death and mortality at work, within one’s family, or related to one’s own life. As several authors have argued, personal goals, values, and sources of meaning in life might also relate to the meanings one ascribes to their death (Cicirelli, 2001; Mikulincer & Florian, 2008; Tomer, 2000; Wong et al., 1994). In addition, demographical characteristics like age and gender might be associated with the presence of certain death meanings (see also Limitations).

As outlined in the introduction, previous findings on the relation between an individual’s worldview and personal meanings of death are scarce or non-existent. However, a comparison between existent empirical investigations pertaining to the Three-Component Model of Death Acceptance (Wong et al., 1994) and our study can be drawn. First, we found the three forms of death acceptance identified by Wong et al. (1994) also in the current analysis: The content category of *salvation and relief* is similar to the construct of escape acceptance, and a *belief in life/existence after death* can be equated with approach acceptance. Based on the corresponding items of the *Death Attitude Profile-Revised* (Wong et al., 1994), neutral acceptance can be regarded as a composite of the concept of *death as something natural and universal* and an *Epicurean view or indifference*. Second, the presence of these death meanings related to an individual’s worldview as follows: A *belief in life/existence after death* correlated positively with religiosity and spirituality but negatively to atheism. Afterlife beliefs were not related to agnosticism. Both *salvation and relief* and *death as something natural and universal* were not associated with any of the four worldview dimensions. An *Epicurean view or indifference* towards one’s own death correlated negatively with religiosity and spirituality, positively with atheism, but not with agnosticism. These results add to the heterogeneous findings for escape and neutral acceptance reported in previous research (Ardelt, 2008; Lin, 2003; Spitzenstätter & Schnell, 2020; Tomer & Eliason, 2005). The relation between these two death attitudes and a person’s worldview, especially religiosity, has thus to be considered unclear—or at least complex—and should be investigated in more detail in future research.

### Synopsis of the Present Study and Previous Research on Death Meanings

The presented approach to investigate views and expectations regarding death differed from previous studies in several ways, foremostly by its method of assessment. To put the results of the present study in a broader context, we conducted an extensive analysis and synopsis of previous quantitative as well as qualitative or theoretical research programs on death meanings and similar constructs (Abdel-Khalek, 2002; Ai et al., 2014; Cicirelli, 1998; 2001; Conte et al., 1982; Diggory & Rothman, 1961; Durlak et al., 1990; Florian & Kravetz, 1983; Fortuin et al., 2021; Hoelter, 1979; Holcomb et al., 1993; Levasseur et al., 2015; Murphy, 1959; Neimeyer et al., 1983; Ross & Pollio, 1991; Spilka et al., 1977; Wong et al., 1994). As displayed in Table C1 and C2 in Supplemental Material C, the list of personal meanings of death derived from the present content analysis can be regarded as the most comprehensive based on its scope. However, the synopsis also points to four aspects that are not adequately represented in this list: First, it might be beneficial to distinguish *positive* (e.g., the outlook of a happy afterlife, see Ai et al., 2014; Wong et al., 1994) from *negative* beliefs in existence or life after death (e.g., punishment in the hereafter, see Abdel-Khalek, 2002; Florian & Kravetz, 1983; Murphy, 1959). This differentiation might be especially important in (more) religious samples. Second, in contrast to the view of death as a motivator and source of meaning in life, one’s own mortality could also be perceived as an *obstacle* for motivation and meaning in life, thus representing a de-motivating, disempowering force and a source of *loss* of meaning in life (see Levasseur et al., 2015). Although this concept was observable in the present sample, its prevalence was so low that it was finally included in the category *other negative associations* and thus did not receive a separate category. Third, the idea of being buried alive or unjustifiably pronounced dead, and fourth, the (imagined) contact with corpses or dead bodies (for both, see Hoelter, 1979), may be regarded as (further) sources of anxiety and fears related to death for certain individuals. Both associations refer to very specific answers to the question of what might be reasons for fearing death—a question that was only indirectly addressed by our stimulus question, which could explain why these concepts did not show up in the present sample. Additionally, contact with corpses or dead bodies does not concern the own death (in a narrow sense) and thus can justifiably be excluded from a list of personal meanings of death.

From a methodological perspective, the DST proved to be a valuable tool for assessing personal meanings of death. It is economical and uncomplicated in its application, sensitive to respondents’ idiosyncratic views and concepts, and brings rich and insightful material to light. In addition, through its non-standardized and open response format, it is perfectly suited for cross-cultural research on death attitudes. With these properties, it should be useful also in psychological counseling and therapy, e.g., as an easy entry for a clinical interview and when a client’s attitudes toward death are of interest. For (nomological) empirical research, the considerable time required for content analysis compared to standardized questionnaires should be kept in mind. Nevertheless, its a priori definition of coding units is a big advantage over less structured qualitative assessments. The coding scheme presented here can be used as a methodological tool for further analyses and research. As implied before, a differentiation between *positive* and *negative* beliefs in life or existence after death as well as a separate category for death as an *obstacle* for motivation and meaning in life could complement the existing list of personal meanings of death.

### Limitations and Future Directions

Several limitations apply to the present study. First, the study was conducted within a specific cultural context that impacts its generalizability and interpretation. On the one hand, prevailing traditions and concepts regarding death and dying—either religiously or societally shaped—might in part have guided the responses of participants. On the other hand, despite methodological rigor and reliability checks for the employed category system, the formation of categories in the course of qualitative content analysis cannot be dissociated from the researchers’ own experiences and prior knowledge, in the current case, especially regarding death and dying. Therefore, the predominantly Catholic, German-speaking and Western-cultural context should be kept in mind when interpreting the present study results. The extent to which the list of death meanings compiled here can be replicated, extended or narrowed down in samples with different religious, cultural, or social backgrounds is a question for future research. However, as our synopsis of previous attempts to assess death meanings could show, our category system is in line and very well compatible with former research.

Next, the sample under investigation consisted of relatively young, highly educated, and three-quarters female participants. Previous research indicated that age and gender are relevant correlates of death anxiety (see, e.g., Neimeyer et al., 2004)—and therefore presumably for personal meanings of death as well. Separate analyses for age and gender were not undertaken due to the small number of older and male participants. Thus, further research is needed to replicate the presented findings in more heterogenous samples or other specific subpopulations.

Lastly, the reported survey was conducted amid the COVID-19 pandemic, an unprecedented event in our generation that might have influenced how individuals thought and felt about their death through an elevated level of mortality salience (Menzies & Menzies, 2020). However, at the time of assessment, incidence rates were relatively low, and several governmental restrictions had been relaxed or repealed in Austria. Therefore, we do not expect too strong biases of the presented results due to the pandemic situation.

### Conclusion

Findings of the present study suggest that the spectrum of concepts, views, and expectations about one’s own death, i.e., personal meanings of death, is broad and multifaceted. Individuals vary greatly in what specific meanings they attribute to their death; relationships to a person’s worldview in this regard are relatively small but existent. Roughly summarized, religious persons show a relatively positive view of death and believe that there is some form of afterlife. The atheists’ perspective is less positive but not necessarily negative and emphasizes that death is the end of existence. Spiritual individuals resemble the religious in several aspects, although they tend to hold less emotionally neutral views toward their death. Agnostics exhibited a generally more negative view of death. The *Death Statements Test* (DST) proved to be a valuable assessment tool to investigate personal meanings of death, worth of further development and future applications.

## Supplemental Material

Supplemental material - The Uncertain Certainty: A Mixed Methods Exploration of Personal Meanings of Death and Preliminary Insights Into Their Relationship With Worldview Supplemental material for The Uncertain Certainty: A Mixed Methods Exploration of Personal Meanings of Death and Preliminary Insights Into Their Relationship With Worldview by Daniel Spitzenstätter and Tatjana Schnell in OMEGA - Journal of Death and Dying

Supplemental material - The Uncertain Certainty: A Mixed Methods Exploration of Personal Meanings of Death and Preliminary Insights Into Their Relationship With Worldview Supplemental material for The Uncertain Certainty: A Mixed Methods Exploration of Personal Meanings of Death and Preliminary Insights Into Their Relationship With Worldview by Daniel Spitzenstätter and Tatjana Schnell in OMEGA - Journal of Death and Dying

Supplemental material - The Uncertain Certainty: A Mixed Methods Exploration of Personal Meanings of Death and Preliminary Insights Into Their Relationship With Worldview Supplemental material for The Uncertain Certainty: A Mixed Methods Exploration of Personal Meanings of Death and Preliminary Insights Into Their Relationship With Worldview by Daniel Spitzenstätter and Tatjana Schnell in OMEGA - Journal of Death and Dying
