# Supplementary material for: The Uncertain Certainty: A Mixed Methods Exploration of Personal Meanings of Death and Preliminary Insights Into Their Relationship With Worldview
Source: Omega (Westport). 2023 Feb 13;91(3):1483–508. doi: 10.1177/00302228231157135 (PMC12188024; doi:10.1177/00302228231157135)
Supplement: Supplemental material - The Uncertain Certainty: A Mixed Methods Exploration of Personal Meanings of Death and Preliminary Insights Into Their Relationship With Worldview [file sj-pdf-1-ome-10.1177_00302228231157135.pdf]

Instruction Text for the Death Statements Test (DST)

Exercise

On the next page, you will find ten blank lines. Please fill them as follows:

- Write in each line a statement or thought that comes to your mind concerning the question **“What does my death mean to me?”**
- Please give your answers in complete sentences.
- In each case, write down what comes to your mind most easily or spontaneously, do not hesitate for long, and continue until you reach the end.
- There are no right or wrong answers in this exercise; only your personal view counts.
- Please work on this exercise “in one go” without interruption. Once you are ready, click on “Proceed.”

[Next page]

Question: What does my death mean to me?

Important:

- One statement or thought in each line
- Answer in complete sentences
- Answer spontaneously and fill in without interruption

01. ....

02. ....

03. ....

04. ....

05. ....

06. ....

07. ....

08. ....

09. ....

10. ....
