# Supplementary material for: The Uncertain Certainty: A Mixed Methods Exploration of Personal Meanings of Death and Preliminary Insights Into Their Relationship With Worldview
Source: Omega (Westport). 2023 Feb 13;91(3):1483–508. doi: 10.1177/00302228231157135 (PMC12188024; doi:10.1177/00302228231157135)
Supplement: Supplemental material - The Uncertain Certainty: A Mixed Methods Exploration of Personal Meanings of Death and Preliminary Insights Into Their Relationship With Worldview [file sj-pdf-2-ome-10.1177_00302228231157135.pdf]

Manual for the Qualitative Content Analysis (Summary)

General Coding Rules

i. Each statement is to be coded regarding its content (33 categories) and its emotional valence (*positive/neutral/negative*).

ii. The content-coding of a single statement should be context-related, i.e., whenever necessary for the understanding of a statement, the remaining statements of a case can be taken into account for interpretation.

Example 1: 1. Death means failure. (= category #4)
2. Everyone fails someday. (= category #14, due to the context of 1.)
3. …

iii. Each statement is assigned exactly one content category. If various issues are thematized in a statement, the category should be coded that is more in focus or emphasized. When this rule is not applicable, category #99 should be coded.

Example 2: "Of course, it is a terrible experience for the dependents, but it is nothing that could be changed in any way."
→ The first clause would suggest category #10, but in the second clause, the inevitability of death is accentuated. Therefore, category #14 is coded.

Example 3: "When I think of death, I think of darkness and loneliness."
→ The statement could be assigned either to category #30 or to category #4. Because the statement as a whole implies something negative, category #4 is coded.

iv. A statement's emotional valence should be rated from the respondent's perspective, not based on the rater's or socially shared values.

Example 4: "My current life is finally over." (= positive)

Table B1

Content Categories

Category	Description	Anchor examples
A. General discomfort with death		
1. Fear of death (unspecified)	Statements expressing a fear of death or death anxiety without further qualifications.	"I fear death."<br>"Death frightens many people."<br>"Anxiety."
2. Loss and transience	Mentions of an unspecified or material loss, of transience, or oblivion.	"Death means loss to me."<br>"You cannot take anything with you."<br>"I will be forgotten."<br>"Someday, no person will be left on earth who knew about my existence."
3. Uncertainty and uncontrollability (non-transcendental)	Associations related to uncertainty regarding the time, the place, the cause, or another aspect related to death, but devoid of any reference to a hereafter or existence after death.	"Something that can happen anytime."<br>"Death entails uncertainty for me."<br>"My death means fear of the uncertain for me."
	Statements that describe death as not controllable, not understandable, or as a loss of power.	"My death means a total loss of control for me."<br>"My death is not understandable for me."
	Mentions that death is final or irreversible.	"Death means finality."
	Ambivalent associations regarding death.	"Death means anxiety as well as calmness for me."
4. Other negative associations	Negative associations regarding death that do not fit into another category.	"Death renders my life meaningless."<br>"Death means despair."<br>"I associate something evil with it."
	All negative associations related to the death of others.	"I am afraid that my beloved ones will die."<br>"The death of others frightens me more than my own death."

Category	Description	Anchor examples
B. General comfort with death		
5. Salvation and relief	Mentions of a (potential or actual) salvation of pain, suffering, stress, sorrows, daily obligations, a meaningless life, etc.	"My death could be a relief after a long disease."<br>"For me, death means salvation from the torments of life."<br>"The end of long working, stress, and demands."
	Mentions of euthanasia or death as a last resort, including suicidal ideations.	"Death should happen self-determined if someone wants to."<br>"Death could be a way out."<br>"Some time ago, it was my wish to die."
6. Peace and calmness	Associations related to peace, calmness, silence, relaxation, or an "eternal sleep."	"I find my peace."<br>"Death means silence to me."<br>"Eternal sleep awaits me."
7. Other positive associations	Positive associations related to death that do not fit into another category.	"My death means gratitude for me."<br>"To be free."
	Statements expressing that an individual does not fear death or does not experience death as something negative.	"My death is nothing negative."<br>"I am not afraid of death."
C. Social consequences of one's own death		
8. Separation from close persons	Mentions of leaving behind or losing close ones or being separated from them.	"Death means to me that I lose my loved ones."<br>"I will not see my friends and family again."<br>"All relationships will end."
9. Inability to be there for others	Mentions that someone cannot provide or be there for close ones anymore, or that close persons have to be left behind and get along on their own.	"When I am no longer alive, I cannot be there for others."<br>"I forsake my family."<br>"It means that my loved ones have to get on without me."

Category	Description	Anchor examples
10. Inconveniences for others	Statements that express emotional, financial, or other inconveniences for dependents related to one's own death.	"My death means grief for my relatives."<br>"My dependents have to pay the funeral costs."<br>"It means much bureaucracy for the dependents."
D. Encounters with death		
11. Death awareness	Associations related to one's own experiences and coping with death, to one's awareness of their mortality, or a call to confront one's own mortality.	"Something that has already come very close to me."<br>"I think about death every day."<br>"Death is omnipresent."<br>"One has to deal with his or her own death."
12. Taboo, repression, avoidance	This category is coded when death is characterized as a societal or personal taboo, when a collective or individual repression of the issue is mentioned, or when someone indicates that they do not confront or confront themselves too less with their own death.	"Death is still a societal taboo in a way."<br>"Something one does not like to talk about."<br>"I repress the thought of death."<br>"My own death is an issue I rarely deal with."
13. Wish for long life or immortality	Coded when a statement refers to a wish for a long(er) life, for immortality, or death at an old age. Additionally, it is coded when the own death is seen as something remote or far away.	"I hope that I will live for a long time."<br>"I would like to become old."<br>"I do not want to die."<br>"Something I would like to prevent."<br>"My death is in the distant future."
E. Rational-analytical considerations on death		
14. Death as something natural and universal	Death is seen as something natural and universal, as a consequence of a natural process, or as a necessary, inevitable part of life. In addition, this category covers mentions related to an acceptance of death or mortality.	"Death is a necessary part of life."<br>"My death simply belongs to the circle of life."<br>"Death is a natural, human event."<br>"Everyone has to die someday."<br>"My death is an inevitable certainty for me."<br>"I accept my mortality."

Category	Description	Anchor examples
15. Epicurean view or indifference	Death is seen as something that does not concern or bother one because death (as a state) cannot be experienced or noticed. This category also covers statements that express indifference toward (one's own) death.	"In the end, my death does not affect me at all because I will never know that I am dead."<br>"As long as I am alive, I should not fear death because I am still alive, and when I am dead, I will not notice it."<br>"My death means nothing to me."<br>"Death is unimportant."
16. Cosmic insignificance	One's own death is seen as insignificant when viewed from the perspective of the universe, the world, or society/humanity as a whole.	"My death means not much to the universe as a whole."<br>"The world will keep turning normally after my death."<br>"My death is one of many."
F. Individual life orientation and evaluation		
17. Death as motivator or source of meaning	Death is seen as a motivating force that influences actions, goals, and priorities in life. Alternatively, an awareness of one's own mortality is associated with existential questions, which might influence one's own view of life and/or evaluation of life as something worthwhile and meaningful.	"The thought of my death reminds me of how important it is to live one's own life."<br>"My death gives meaning to my life."<br>"The prospect of my death gives me perspective and the necessary motivation to achieve my goals."<br>"My death allows me to value my life and time."
18. Death as a trial	One's own death is associated with a time of reckoning, with a final reflection on one's life, or with a task that has to be accomplished.	"My death brings the reckoning for all my good and bad deeds."<br>"The moment I see which kind of person I was."<br>"To draw a conclusion."<br>"Death is the last opponent that has to be defeated."

Category	Description	Anchor examples
19. Termination of plans/goals/possibilities	Death ends one's commenced projects and hinders one from accomplishing their goals in life. Death is also the end of the possibility to gather new experiences or to undo or change something that has been done previously.	"I can't do anymore what I wanted to do but haven't done yet."<br>"A sudden death would end my future plans, which I would regret."<br>"Death makes all irrevocable, and failures cannot be corrected anymore."<br>"My death is the end of my possibilities."
20. Generativity and remembrance	Statements that express that one wants to be remembered and/or wants to leave enduring traces in the world.	"I want to be remembered."<br>"I want to leave traces of joy and love."<br>"I have to pay attention to what I will bequeath."<br>"I hope that others will miss me."<br>"I do not want to die without having children."
21. Wish for a positive life review	Implicit or explicit wish or conviction that one has used their lifetime well, that goals were accomplished, that there is nothing to regret, and/or that one can be satisfied with their life at the time of death.	"Hopefully, I have lived a beautiful life."<br>"I want to have lived a fulfilled life."<br>"Hopefully, I do not regret anything."<br>"I am convinced that I will be satisfied with my life at the time of my death."<br>"I was not able to do something useful with my life."
G. Postmortem (non-)existence		
22. Death as the end (unspecified)	Death is associated with an end that is not specified further.	"Death is the end."<br>"Death means a closing for me."
23. Death as the end of physical functions	Death is characterized as the end of specific or all physical functions and/or the beginning of bodily decay.	"The end of the vital functions of my body."<br>"My body will rot."<br>"Reorganization of my cells, atoms, etc."
24. Death as the end of existence	Death is associated with the (complete) end of one's existence, one's self, and/or one's being.	"The end of my existence."<br>"My death means my end."<br>"I do not believe in a soul."<br>"Everything will be over."

Category		Description	Anchor examples
25.	Death as the end of life	Death is characterized as the end of life, devoid of further explanations whether this means a complete or only a partial end of the own existence.	"The end of my life."<br>"When I am dead, I am no longer alive."<br>"Death means the loss of life."
26.	Death as the end of mental abilities	Death is described as the end of specific mental abilities (thoughts, feelings, memory, …) or as the end of consciousness.	"I cannot think anymore."<br>"I do not have thoughts or feelings anymore."<br>"I lose my consciousness through my death."
27.	Belief in life/existence after death	A belief or conviction that there is some form of life or existence after death or that death entails a transition to something other or new.	"After death, there is life."<br>"My death is the beginning of another form of existence."<br>"The transition to a new, other world."<br>"To see already deceased and missed persons again."
28.	Postmortem uncertainty	Uncertainty or indecision regarding life or existence after death, or fears related to the unknown after death.	"I wonder how it will go on afterward."<br>"I am afraid that it won't go on after death."<br>"I don't know what will happen after death."<br>"I wish I could believe in a life after death."
29.	Hope for life/existence after death	A hope—but not a belief or conviction—for some form of life or existence after death is expressed.	"I hope for a life after death."<br>"I hope to see my dad again."<br>"Maybe there is an afterlife."
H. Dying and the ending of life			
30.	Death as an experiential state	Death is associated with a sensory perception or some form of experiential state.	"Death means darkness for me."<br>"It means coldness for me."<br>"A new and intense experience."<br>"I imagine my death as dark, cotton-soft, and mysterious."

Category	Description	Anchor examples
31. Thoughts on funeral/burial	Thoughts and associations related to one's funeral, the form of burial, mourning ceremony, or treatment of one's corpse.	"I want to be cremated after my death."<br>"I hope the mourning ceremony will be beautiful."<br>"I wonder who will attend my funeral."<br>"My body is still intimate when it ceases to breathe."
32. Thoughts on the dying process	All associations related to the process of dying or how it feels like to die not referring to a wish for a "good" death/dying.	"Will dying be painful?"<br>"What do you think about when you are dying?"<br>"I get depressive when I think of my dying."<br>"I am afraid of dying."
33. Wish for a "good" death	Wish for a painless and peaceful death/dying without suffering and with the opportunity to take farewell from and be with close persons at the time of death.	"It should be a natural death."<br>"I hope that my death is peaceful and beautiful, not painful."<br>"I want to say goodbye to all my loved ones before I die."<br>"I would like to pass away gently."<br>"I want to die with dignity."
X. Residual categories		
99. Not assignable	Statements that do not fit into categories #1 to #33 or could not be assigned to one single category but represent a meaningful response.	"God loves me."<br>"I am dead."
999. Not codable	Statements that are not meaningful responses.	"#+$%&."<br>"(I have no more ideas)."

Table B2

Emotional Valence Rating Categories

Valence		Description	Anchor examples
-1	Negative	Statements that clearly express negative attitudes, feelings, or changes related to one's own death/dying; or statements that express something unwanted with regard to one's own death/dying; or when a positive valence of death is negated.	"I fear death."<br>"Death renders my life meaningless."<br>"Death means to me that I lose my loved ones."<br>"I do not want to die."<br>"In any case, death is nothing positive."
0	Neutral	Statements that neither express a negative nor a positive valence for the respondent; or statements where an emotional valence cannot be clearly deduced or is ambivalent; or statements that express questions, hopes, or wishes.	"Death is a necessary part of life."<br>"I do not believe in a soul."<br>"Death means anxiety as well as calmness for me."<br>"Will dying be painful?"<br>"I hope for a life after death."
+1	Positive	Statements that clearly express positive attitudes, feelings, or changes related to one's own death/dying; or when a negative valence of death is negated.	"My death means gratitude for me."<br>"For me, death means salvation from the torments of life."<br>"My death is nothing negative."
