# Supplementary material for: The Uncertain Certainty: A Mixed Methods Exploration of Personal Meanings of Death and Preliminary Insights Into Their Relationship With Worldview
Source: Omega (Westport). 2023 Feb 13;91(3):1483–508. doi: 10.1177/00302228231157135 (PMC12188024; doi:10.1177/00302228231157135)
Supplement: Supplemental material - The Uncertain Certainty: A Mixed Methods Exploration of Personal Meanings of Death and Preliminary Insights Into Their Relationship With Worldview [file sj-pdf-3-ome-10.1177_00302228231157135.pdf]

Table C1

Synopsis of quantitative instruments assessing various death attitudes/meanings and the present system of personal meanings of death.

Instrument	Subscale	Fear of death (unspecified)	Loss and transience	Uncertainty and uncontrollability (non-transc.)	Other negative associations	Salvation and relief	Peace and calmness	Other positive associations	Separation from close persons	Inability to be there for others	Inconveniences for others	Death awareness	Taboo, repression, avoidance	Wish for long life or immortality	Death as something natural and universal	Epicurean view or indifference	Cosmic insignificance	Death as motivator or source of meaning	Death as a trial	Termination of plans/goals/possibilities	Generativity and remembrance	Wish for a positive life review	Death as the end (unspecified)	Death as the end of physical functions	Death as the end of existence	Death as the end of life	Death as the end of mental abilities	Belief in life/existence after death	Postmortem uncertainty	Hope for life/existence after death	Death as an experiential state	Thoughts on funeral/burial	Thoughts on the dying process	Wish for a “good” death	(Not covered by present content categories)
Death Perspectives Scale (DPS; Spilka et al., 1977)	1. Death as Pain and Loneliness				X	X																											X		
	2. Death as an Afterlife-of-Reward																											X							
	3. Indifference toward Death															X																			
	4. Death as Unknown			X																									X						
	5. Death as Forsaking Dependents plus guilt				X					X																									
	6. Death as Courage																		X																
	7. Death as Failure																			X															
	8. Death as a Natural End														X																				
Personal Meanings of Death Scale (PMDS; Cicirelli, 1998, 2001)	1. Legacy							X											X		X														
	2. Afterlife																											X							
	3. Extinction		X		X				X											X					X										
	4. Motivator																	X																	
Reasons for Death Fear Scale (RDFS; Abdel-Khalek, 2002)	1. Fear of Punishment & Pain			X	X																							X	X				X		
	2. Fear of Losing Worldly Involvement		X																	X					X										
	3. Religious Transgressions & Failures				X																														X
	4. Parting from Loved Ones								X	X	X																								
Multidimensional Fear of Death Scale (MFODS; Hoelter, 1979)	1. Fear of the Dying Process																																X		
	2. Fear of the Dead				X																														X
	3. Fear of Being Destroyed																															X			
	4. Fear of Significant Others				X						X																								
	5. Fear of the Unknown				X																							X	X						
	6. Fear of Conscious Death																														X	X			X
	7. Fear of the Body after Death																							X								X			
	8. Fear of Premature Death																			X															

Instrument	Subscale	Fear of death (unspecified)	Loss and transience	Uncertainty and uncontrollability (non-transc.)	Other negative associations	Salvation and relief	Peace and calmness	Other positive associations	Separation from close persons	Inability to be there for others	Inconveniences for others	Death awareness	Taboo, repression, avoidance	Wish for long life or immortality	Death as something natural and universal	Epicurean view or indifference	Cosmic insignificance	Death as motivator or source of meaning	Death as a trial	Termination of plans/goals/possibilities	Generativity and remembrance	Wish for a positive life review	Death as the end (unspecified)	Death as the end of physical functions	Death as the end of existence	Death as the end of life	Death as the end of mental abilities	Belief in life/existence after death	Postmortem uncertainty	Hope for life/existence after death	Death as an experiential state	Thoughts on funeral/burial	Thoughts on the dying process	Wish for a “ good” death	(Not covered by present content categories)
Fear of Personal Death scale (FPD; Florian & Kravetz, 1983)	1. Loss of Self-Fulfillment		X						X									X		X		X				X	X								
	2. Self-Annihilation						X																	X	X		X								
	3. Loss of Social Identity		X														X							X								X			
	4. Consequences to Family & Friends									X	X																								
	5. Transcendental Consequences			X																									X						
	6. Punishment in the Hereafter				X																							X	X						X
Interpretation of Death scale (IOD; Fortuin et al. 2021)	1. Religious Interpretation of Death																		X									X							
	2. Personal Interpretation of Death							X													X	X										X		X	
	3. Rational Interpretation of Death																								X		X						X		
Multidimensional Mortality Awareness Measure (MMA-M; Levasseur et al., 2015)	1. Mortality legacy																				X														
	2. Mortality fearfulness			X	X							X	X	X						X															
	3. Mortality acceptance							X							X																				
	4. Mortality disempowerment			X	X												X																		X
	5. Mortality disengagement							X					X																						
Death Anxiety Questionnaire (DAQ; Conte et al., 1982)	1. Fear of the Unknown								X											X					X				X				X		
	2. Fear of Suffering																																X		
	3. Fear of Loneliness																																	X	
	4. Fear of Personal Extinction																				X														X
	5. (not interpretable)										X																						X		
Connection of Soul scale (COS; Ai et al., 2014)	1. Secular view																								X										
	2. God-centered view																											X							
	3. Cosmic-spiritual view																											X							
Death Attitude Profile–Revised (DAP-R; Wong et al. 1994)	1. Fear of death	X	X	X	X																								X						
	2. Death avoidance												X																						
	3. Neutral Acceptance														X	X																			
	4. Approach Acceptance																											X							
	5. Escape Acceptance					X																													

Note. Assignments are based on item formulations; a subscale might thus correspond to multiple subjective death meanings. Full references can be found in the main document.

Table C2

Synopsis of qualitatively and/or theoretically derived lists of death meanings and the present system of personal meanings of death.

Author(s)		Category/Dimension	Fear of death (unspecified)	Loss and transience	Uncertainty and uncontrollability (non-transc.)	Other negative associations	Salvation and relief	Peace and calmness	Other positive associations	Separation from close persons	Inability to be there for others	Inconveniences for others	Death awareness	Taboo, repression, avoidance	Wish for long life or immortality	Death as something natural and universal	Epicurean view or indifference	Cosmic insignificance	Death as motivator or source of meaning	Death as a trial	Termination of plans/goals/possibilities	Generativity and remembrance	Wish for a positive life review	Death as the end (unspecified)	Death as the end of physical functions	Death as the end of existence	Death as the end of life	Death as the end of mental abilities	Belief in life/existence after death	Postmortem uncertainty	Hope for life/existence after death	Death as an experiential state	Thoughts on funeral/burial	Thoughts on the dying process	Wish for a " good" death	(Not covered by present content categories)
Murphy (1959)	1.	Death is the end																					X												
	2.	Fear of losing consciousness			X																							X								
	3.	Fear of loneliness								X																					X					
	4.	Fear of the unknown			X																									X						
	5.	Fear of punishment			X															X									X	X						X
	6.	Fear of what may happen to one's dependents									X	X																								
	7.	Fear of failure																			X		X													
Diggory & Rothman (1961)	A.	I could no longer have any experiences																			X							X								
	B.	I am uncertain as to what might happen to me if there is life after death																												X						
	C.	I am afraid of what might happen to my body after death																															X			
	D.	I could no longer care for my dependents									X																									
	E.	My death would cause grief to my relatives and friends										X																								
	F.	All my plans and projects would come to an end																			X															
	G.	The process of dying might be painful																																X		
Durlak et al. (1990)	1.	Religious Outlook																											X							
	2.	Termination of Experiences				X															X															
	3.	Impact on Others									X	X																								
	4.	Continued Existence																											X							
	5.	Uncertainty			X																									X						
	6.	Favorable							X																											
	7.	Inevitability														X									X											

Author(s)		Category/Dimension	Fear of death (unspecified)	Loss and transience	Uncertainty and uncontrollability (non-transc.)	Other negative associations	Salvation and relief	Peace and calmness	Other positive associations	Separation from close persons	Inability to be there for others	Inconveniences for others	Death awareness	Taboo, repression, avoidance	Wish for long life or immortality	Death as something natural and universal	Epicurean view or indifference	Cosmic insignificance	Death as motivator or source of meaning	Death as a trial	Termination of plans/goals/possibilities	Generativity and remembrance	Wish for a positive life review	Death as the end (unspecified)	Death as the end of physical functions	Death as the end of existence	Death as the end of life	Death as the end of mental abilities	Belief in life/existence after death	Postmortem uncertainty	Hope for life/existence after death	Death as an experiential state	Thoughts on funeral/burial	Thoughts on the dying process	Wish for a " good" death	(Not covered by present content categories)
Ross & Pollio (1991)	I-A	Negatively: Barrier/Limit to being (in general)																								X										
	I-B	Negatively: Barrier/Limit to engaging with others								X	X																									
	I-C	Negatively: Barrier/Limit to accomplishing new projects		X																	X															
	I-D	Negatively: Barrier/Limit to control/predictability			X																															
	I-E	Negatively: Barrier/Limit to understanding (being able to conceptualize)			X																							X								
	I-F	Negatively: Barrier/Limit to body vitality																							X											
	II-A	Ambivalent: Negative, but acceptable because of the presence of loved ones																																	X	
	II-B	Ambivalent: Negative, but acceptable because of accomplishments/fulfillments																					X													
	II-C	Ambivalent: Negative, but acceptable because of being part of a natural event														X																				
	II-D	Ambivalent: Negative, but acceptable because of the escape from pain/sickness					X																													
	III-A	Positively: Transformation (symbolic immortality)																				X														
	III-B	Positively: Transformation (afterlife)																											X							
	III-C	Positively: Transformation (growth through confrontation)											X						X																	

Author(s)		Category/Dimension	Fear of death (unspecified)	Loss and transience	Uncertainty and uncontrollability (non-transc.)	Other negative associations	Salvation and relief	Peace and calmness	Other positive associations	Separation from close persons	Inability to be there for others	Inconveniences for others	Death awareness	Taboo, repression, avoidance	Wish for long life or immortality	Death as something natural and universal	Epicurean view or indifference	Cosmic insignificance	Death as motivator or source of meaning	Death as a trial	Termination of plans/goals/possibilities	Generativity and remembrance	Wish for a positive life review	Death as the end (unspecified)	Death as the end of physical functions	Death as the end of existence	Death as the end of life	Death as the end of mental abilities	Belief in life/existence after death	Postmortem uncertainty	Hope for life/existence after death	Death as an experiential state	Thoughts on funeral/burial	Thoughts on the dying process	Wish for a "good" death	(Not covered by present content categories)
Neimeyer et al. (1984), Holcomb et al. (1993)	1a.	Purposeful																	X																	
	1b.	Purposeless				X												X																		
	2a.	Positive evaluation							X																											
	2b.	Negative evaluation				X																														
	3b.	Negative emotional state				X																														
	4b.	Low acceptance												X																						
	5a.	High understanding											X																							
	5b.	Low understanding			X																															
	6a.	High Suffering				X																														
	6b.	Low Suffering							X																											
	7a.	High personal involvement											X																							
	7b.	Low personal involvement												X	X																					
	8a.	Long range temporal expectation											X																							
	8b.	Short range temporal expectation			X																															
	9a.	High certainty														X																				
	10a.	Existence																											X							
	10b.	Nonexistence																								X										
	11a.	High choice					X																													
	11b.	Low choice			X																															
	12a.	Specific											X																							
	12b.	General														X																				
	13a.	High impact				X															X															
	13b.	Low impact															X																			
	14a.	Known causality											X												X											
	14b.	Unknown causality			X																															
	15.	Wishful thinking			X										X								X							X	X				X	

Note. Assignments are based on definitions and explanations from the cited authors. Full references can be found in the main document. Categories 3a, 4a, and 9b of Neimeyer et al. (1984) and Holcomb et al. (1993) were eliminated by the authors due to unreliability (see Neimeyer et al., 1984).
